# Supplementary figures and images for: QTL analysis of traits related to seed size and shape in sesame (Sesamum indicum L.)
Source: PLoS One. 2023 Nov 2;18(11):e0293155. doi: 10.1371/journal.pone.0293155 (PMC10621824; doi:10.1371/journal.pone.0293155)

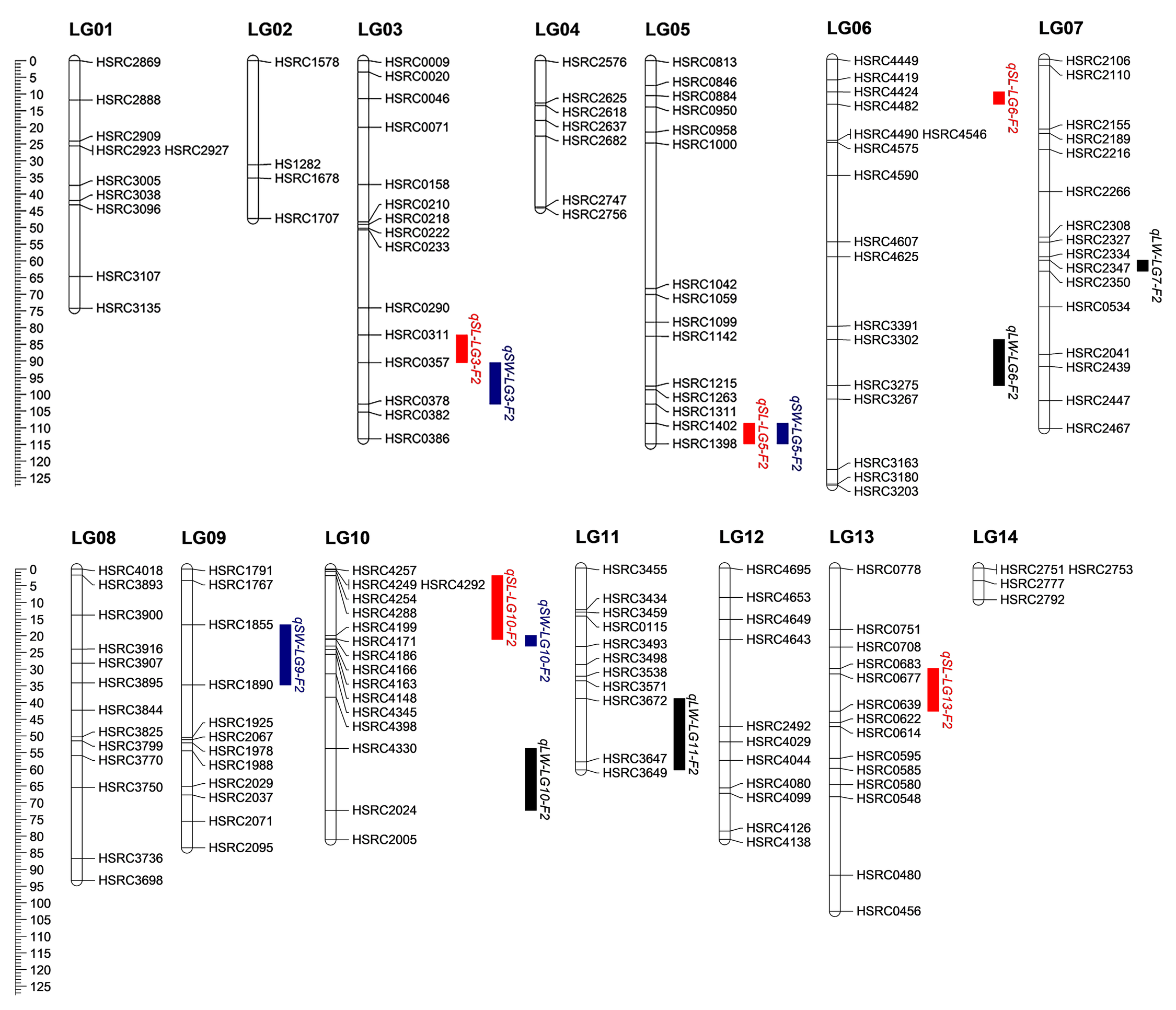

Supplement: S1 Fig — The black bar on each LG column indicates a SSR marker. The QTLs for SL, SW, and L/W are shown in red, blue, and black, respectively. (TIF) [file pone.0293155.s001.tif]

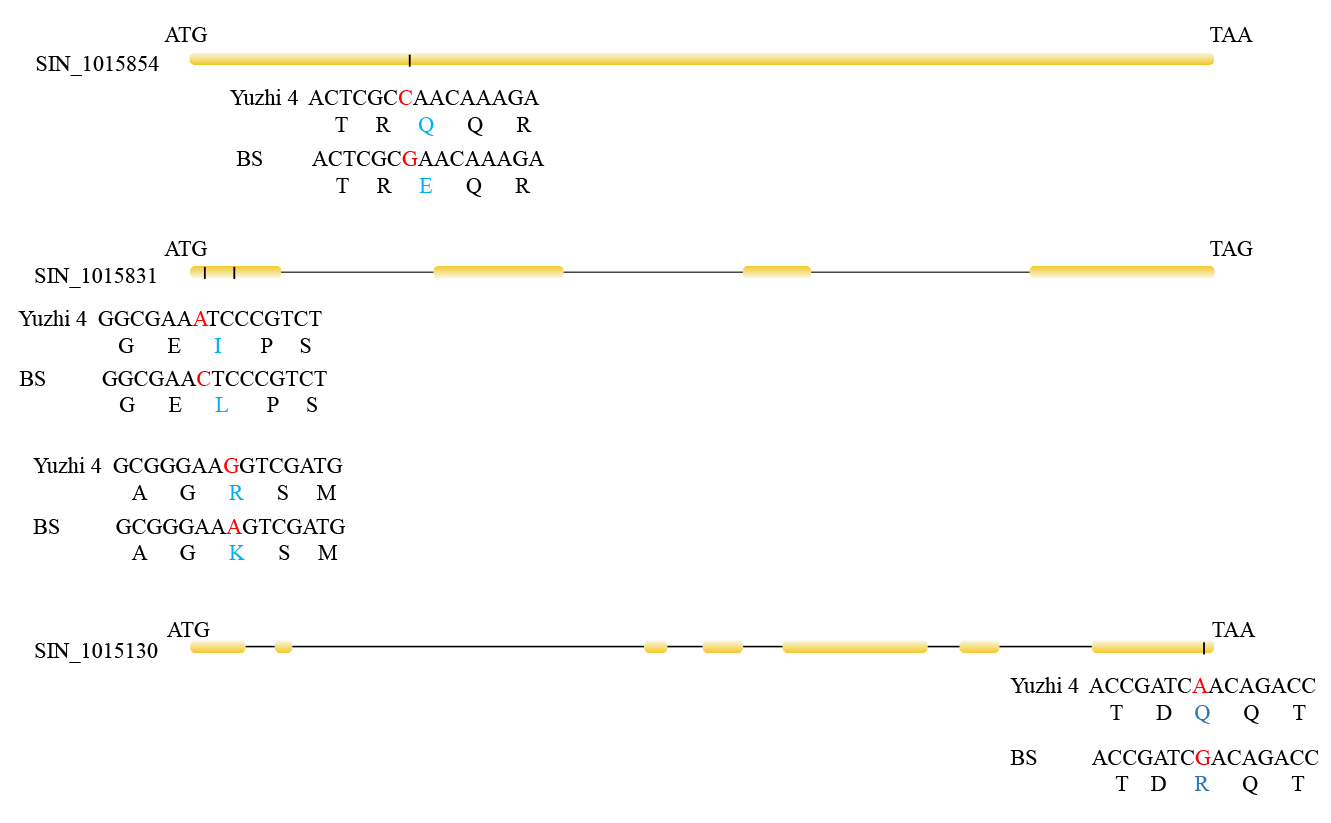

Supplement: S2 Fig — (TIF) [file pone.0293155.s002.tif]

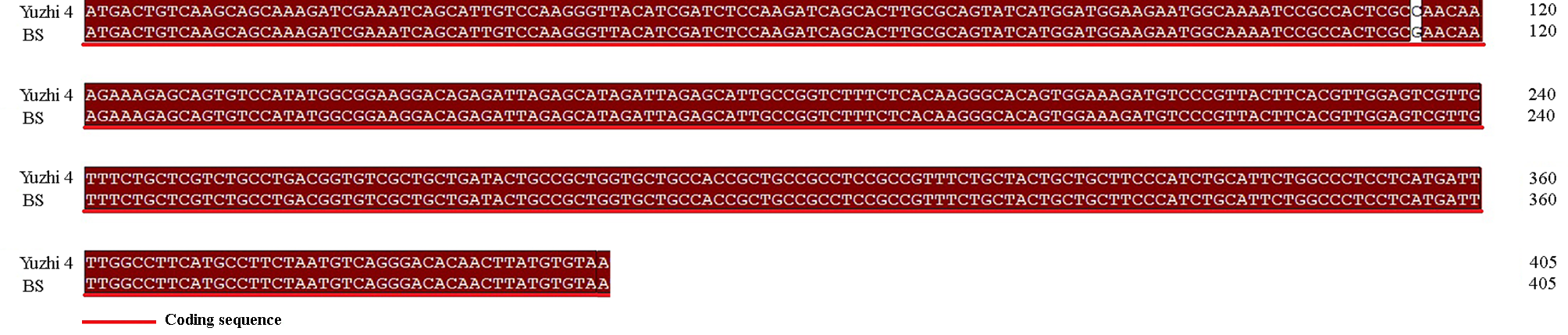

Supplement: S3 Fig — (TIF) [file pone.0293155.s003.tif]

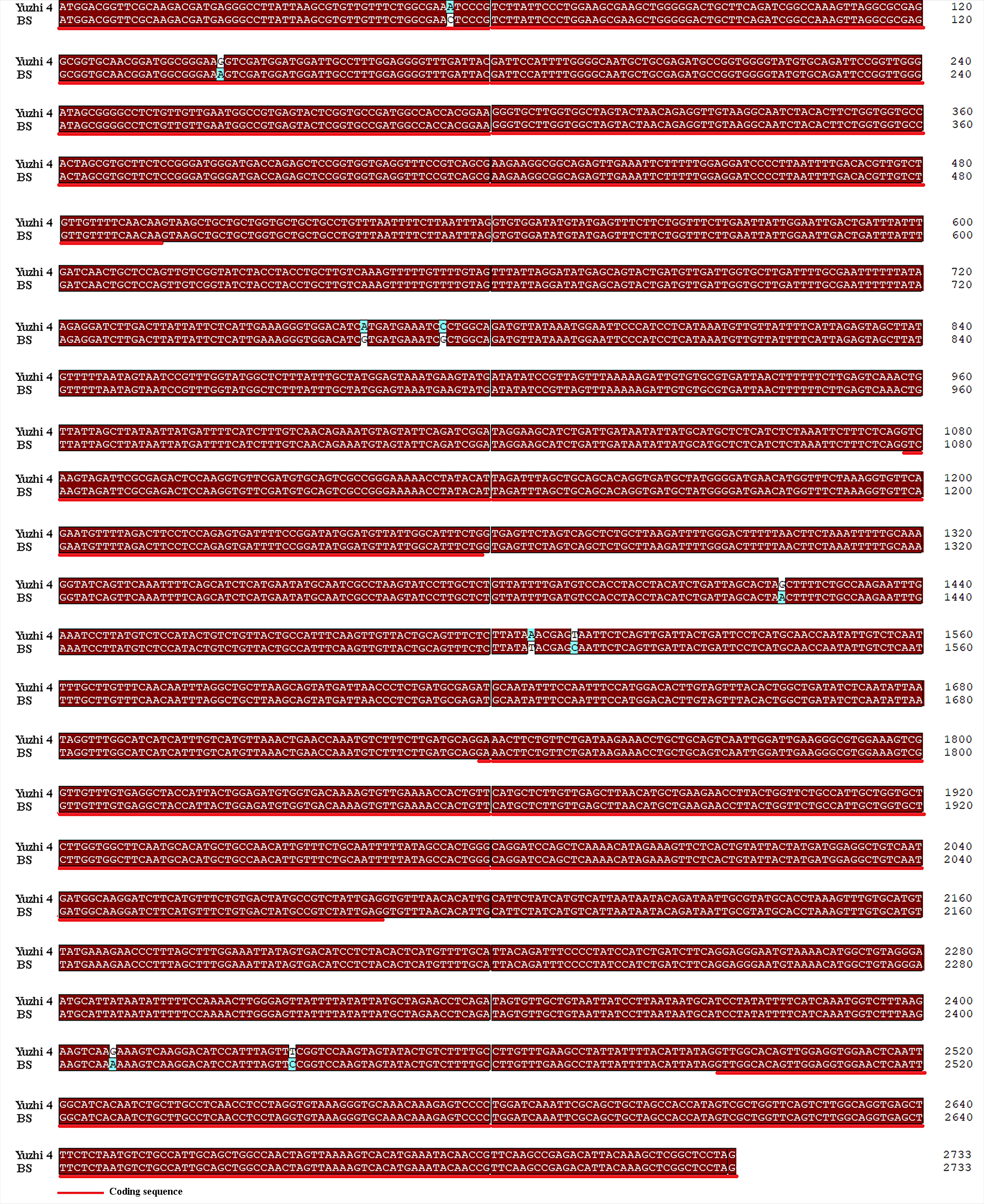

Supplement: S4 Fig — (TIF) [file pone.0293155.s004.tif]

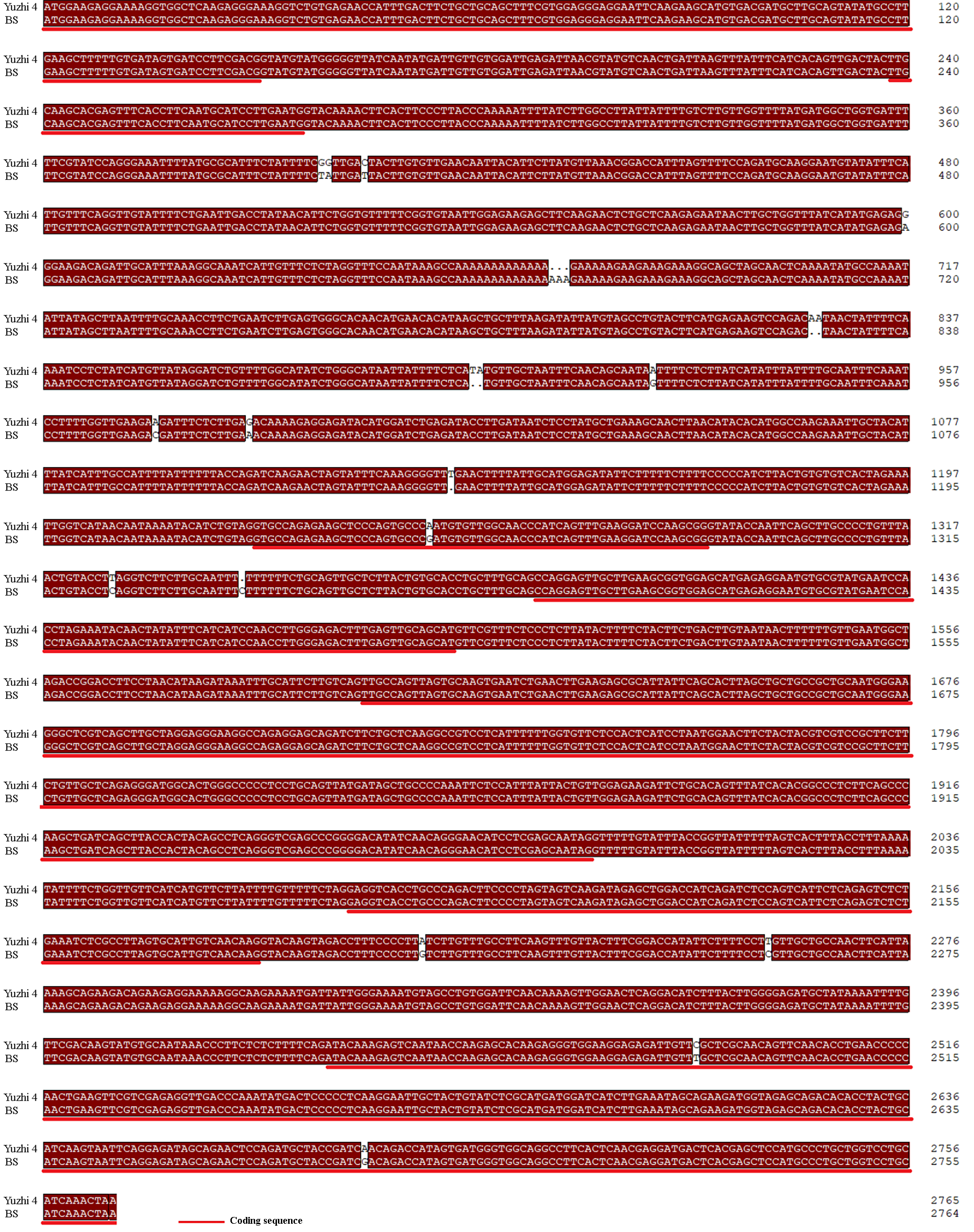

Supplement: S5 Fig — (PNG) [file pone.0293155.s005.png]
