# Supplementary material for: QTL analysis of traits related to seed size and shape in sesame (Sesamum indicum L.)
Source: PLoS One. 2023 Nov 2;18(11):e0293155. doi: 10.1371/journal.pone.0293155 (PMC10621824; doi:10.1371/journal.pone.0293155)
Supplement: Attachment — Submitted filename: Response to reviewers.docx [file pone.0293155.s013.docx]

Thank you for the reviewers’ comments concerning our manuscript entitled “QTL analysis of traits related to seed size and shape in sesame (*Sesamum indicum* L.)”. Those comments are all valuable and very helpful for revising and improving our paper, as well as the important guiding significance to our research. We have studied comments carefully and have made correction which we hope meet with approval. Revised manuscript has been uploaded. The main corrections in the paper and the responds to the reviewer’s comments are as flowing:

Reviewer #1: The Manuscript provides information on QTLs linked to sesame seed size and shape, which is a rare report in the public. However, there were still some minor concerns in the study.

**1. Line 70,72 “SNP” should be “SNPs”.**

We are grateful for the suggestion. Yes，“SNP” has been amended to “SNPs”

**2. Line 133, “QTLs with more than 10% of PVE regarded as major QTLs”, What is the basis for this regarded?**

Mei et al (2021) reported that QTLs that explained more than 10% of phenotypic variation in at least one environment were considered major QTLs in the materials and methods. In results, they performed QTL mapping for yield‑related traits in sesame, and identified 20 major QTLs that explained more than 10% of the corresponding phenotypic variation in at least one environment. We added this reference in line 133.

Mei H, Liu Y, Cui C, Hu C, Xie F, Zheng L, et al. QTL mapping of yield‑related traits in sesame. Mol Breeding. 2021; 41(7):43. doi:10.1007/s11032-021-01236-x.

**3. Line 260, “sequence analysis detected” is cloning in both parents? If so, the sequencing results are included** **as Supporting information in the manuscript.**

Yes, the genes were cloned and sequenced in both parents. The sequencing results were provided as Supporting information (S3-S5 Figs).

**4. The formatting of the references needs to be revised. Add units for traits in Table 1****. How identification of** **the sesame seed size and shape QTLs could be useful in future molecular breeding program?**

Thank you for the suggestion. We have revised the formatting of the references and added units for traits in Table 1 (line 162). In this study, we identified several sesame seed size and shape QTLs. We will develop molecular markers closely linked to the related traits based on the QTLs, and use these markers to select objective trait. Furthermore, candidate genes and causative sites for these important traits will be identified by QTL fine-mapping or GWAS in sesame. With more genes that underlie quantitative traits identified, navigation breeding will be applied in sesame, which has been successfully used in rice (Wei et al. 2021).

Wei X, Qiu J, Yong K, Fan J, Zhang Q, Hua H, et al. A quantitative genomics map of rice provides genetic insights and guides breeding. Nat Genet. 2021; 53(2):243-253. doi:10.1038/s41588-020-00769-9.

**5. Based on the gene mapping and QTL analysis, authors found three candidate genes for seed size and shape. Do you have any plan for the complementation study to confirm the gene function？**

Yes, we will analyze the expression patterns of three candidate genes using qRT-PCR, analyze the location of the proteins by subcellular localization, and verify the function of the genes using genetic transformation technology.

Reviewer #2:

**1. in line 146，‘the seed size of the BS line was smaller’, there should be added statistical analysis for the phenotype of the parents.**

Thanks for your suggestion. Yes, statistical analysis for the phenotype of the parents is shown in Figure 1B.

**2. in line 169-171, ‘the phenotypic values of the three traits were significantly positively correlated between environments in the BC1 population.’ The wording here is unclear.**

We are very sorry for our unclear description. This sentence has been rephrased as follows: for each of the traits, a significantly positive correlation of the phenotypic values between each of environments in the BC1 population. (Line 170-171)

**3. the description of linkage map should be added in the results.**

Yes, we have added the description of linkage map in manuscript. (Line 185-187; line 220)

**4 in line 260-262, non-synonymous SNPs were identified, maybe we can use software to predict whether the SNPs affect the protein function, http://www.ppved.org.cn/index.html. The transcription level of the candidate genes also should be** **considered.**

Yes, we have performed prediction of protein function by the software, and found that SNPs did not affect the protein function. Therefore, we will perform the qRT-PCR to consider the transcription level of the candidate genes, and carry out the subcellular localization and genetic transformation to analyze function of the candidate genes in next steps.

We tried our best to improve the manuscript and made some changes in the manuscript. These changes will not influence the content and framework of the paper. We appreciate for Editors/Reviewers’ warm work earnestly, and hope that the correction will meet with approval.

Once again, thank you very much for your comments and suggestions.

Sincerely yours

Yongzhan Zheng
